# Augmenting Human Selves Through Artificial Agents – Lessons From the Brain

**DOI:** 10.3389/fncom.2022.892354

**Published:** 2022-06-23

**Authors:** Georg Northoff, Maia Fraser, John Griffiths, Dimitris A. Pinotsis, Prakash Panangaden, Rosalyn Moran, Karl Friston

**Affiliations:** ^1^Mental Health Center, Zhejiang University School of Medicine, Hangzhou, China; ^2^Department of Mind, Brain Imaging and Neuroethics, Institute of Mental Health Research, University of Ottawa, Ottawa, ON, Canada; ^3^Centre for Research Ethics & Bioethics, Uppsala University, Uppsala, Sweden; ^4^Department of Mathematics and Statistics, University of Ottawa, Ottawa, ON, Canada; ^5^Centre for Addiction and Mental Health (CAMH), Toronto, ON, Canada; ^6^Department of Psychiatry, University of Toronto, Toronto, ON, Canada; ^7^Centre for Mathematical Neuroscience and Psychology, Department of Psychology, City, University of London, London, United Kingdom; ^8^The Picower Institute for Learning and Memory, Massachusetts Institute of Technology, Cambridge, MA, United States; ^9^Department of Computer Science, McGill University, Montreal, QC, Canada; ^10^Montreal Institute for Learning Algorithms (MILA)., Montreal, QC, Canada; ^11^Centre for Neuroimaging Sciences, Institute of Psychiatry, Psychology and Neuroscience, King’s College London, London, United Kingdom; ^12^Wellcome Centre for Human Neuroimaging, London, United Kingdom; ^13^Institute of Neurology, University College London, London, United Kingdom

**Keywords:** intelligence augmentation (IA), spatio – temporal dynamics, free energy principle, free energy principle and active inference (FEP-AI) framework, human self, hierarchical learning, agent-environment interaction

## Abstract

Much of current artificial intelligence (AI) and the drive toward artificial general intelligence (AGI) focuses on developing machines for functional tasks that humans accomplish. These may be narrowly specified tasks as in AI, or more general tasks as in AGI – but typically these tasks do not target higher-level human cognitive abilities, such as consciousness or morality; these are left to the realm of so-called “strong AI” or “artificial consciousness.” In this paper, we focus on how a machine can *augment* humans rather than *do* what they do, and we extend this beyond AGI-style tasks to augmenting peculiarly personal human capacities, such as wellbeing and morality. We base this proposal on associating such capacities with the “self,” which we define as the “environment-agent nexus”; namely, a fine-tuned interaction of brain with environment in all its relevant variables. We consider richly adaptive architectures that have the potential to implement this interaction by taking lessons from the brain. In particular, we suggest conjoining the free energy principle (FEP) with the dynamic temporo-spatial (TSD) view of neuro-mental processes. Our proposed integration of FEP and TSD – in the implementation of artificial agents – offers a novel, expressive, and explainable way for artificial agents to adapt to different environmental contexts. The targeted applications are broad: from adaptive intelligence augmenting agents (IA’s) that assist psychiatric self-regulation to environmental disaster prediction and personal assistants. This reflects the central role of the mind and moral decision-making in most of what we do as humans.

*“We are like islands in the sea, separate on the surface but connected in the deep”*

*(William James)*

## Introduction: Aim – Augmenting Human Intelligence

### From Environment to Agents – Lessons From the Brain

The ambitious goal of artificial general intelligence (AGI) is often stated as building machines that can perform any intellectual task a human can. While this is still out of reach, and somewhat vague, current artificial intelligence (AI) has already reached and sometimes superseded human abilities on many narrowly defined tasks, such as game-playing and image analysis. Current approaches to AGI often focus on extending this ability to less narrowly defined environments and more complex or partly novel tasks. In contrast, explicitly human capacities such as morality or consciousness are typically considered outside the purview of AGI. Although they are of importance in Cognitive Science and Philosophy, much of mainstream Computer Science has abandoned their pursuit.

In this paper, we focus on how a machine can *augment* humans rather than *do* what they do, and we extend this beyond AGI-style tasks. We argue that augmentation is possible even for very personal human capacities such as wellbeing and morality. We base augmentation on how these capacities link to the *self*, which we interpret in a broad but technical sense as the brain’s “environment-agent nexus”: that is, the fine-tuned interaction of the individual’s brain with – and its alignment to – the environment, including the full gamut of sensory, social and cultural features of the environment (Northoff and Stanghellini, 2016; Northoff, 2018a; Constant et al., 2020; Scalabrini et al., 2020). The relevance of such environment-agent nexus becomes particularly visible in times of changing environmental contexts as during pandemics like COVID as they strongly impact the agent, i.e., our self (Scalabrini et al., 2020).

Our proposal targets the functionality of the human environment-agent nexus and, specifically, its potential augmentation by a machine. For an artificial agent to assist in the regulation of such a delicate interplay, great sensitivity and adaptivity of its own agent-environment nexus will be required – if not at a human level, then at least a more refined level than current artificial agents. We argue that lessons from the brain hold great promise for modeling and implementing this.

### Building More Adaptive Agents – Conjoining Free Energy and Temporo-Spatial Dynamics

More precisely, as an agnostic and flexible approach to building richly aligning agents, in this paper we hypothesize that conjoining the free energy principle with the dynamic temporo-spatial view of neuro-mental processes (Friston et al., 2006; Northoff et al., 2020) offers a promising avenue. Some key observations about the brain motivate this.

First, the brain exists in a temporally continuous interface with the environment, which has been described in terms of the free energy principle (FEP). At the core of the FEP is Variational Free Energy, which is computed given states of two systems: the agent and its environment. In our proposal, we propose to apply it to the temporo-spatial dynamics (TSD) of agent and environment. Crucially, the brain’s TSD is organized in a hierarchical manner, according to time and space scales, which are adaptively determined and finely nested (as described in detail in see section “Intrinsic Organization of the Brain – Spatial and Temporal Hierarchies5”). This temporo-spatial hierarchy has been associated with neuro-mental processes for both self and consciousness (Tagliazucchi et al., 2013, 2016; Huang et al., 2016, 2018; Zhang et al., 2018; Wolff et al., 2019a; Northoff et al., 2020).

Our core proposal is that equipping artificial agents with hierarchical, free energy minimizing temporo-spatial dynamics could be crucial for improving their ability to align to changing environmental contexts, and – in particular – to dyadic exchanges with humans. We anticipate that our agents may augment human capabilities by being able to access or observe the environment in ranges that far exceed those of humans, either by accumulating experience over many human-years or measuring quantities we cannot observe. Nevertheless, these agents should necessarily align with the environment with which they exchange, which includes the humans that they should augment.

In the remainder of this paper, after an interlude to sketch possible future scenarios, we discuss the road toward adaptive agents in see section “Artificial Intelligence and Environment – Learning From the Brain’s Adaptive Capacities,” then focus on two key lessons from the brain: the conjoining of FEP and TSD in see section “Environment-Brain Interface – Conjoining the Free Energy Principle and Temporo-Spatial Dynamics,” and spatial- and temporal hierarchy in see section “Intrinsic Organization of the Brain – Spatial and Temporal Hierarchies”. See section “Modeling the Environment-Agent Nexus Using Free Energy Principle and Temporo-Spatial” will pick up the modeling of artificial agents and what can be learned from the brain, while see section “Can Artificial Agents Augment Humans – Coming Back to Our Examples” will return to our examples and how our novel AI models may address the issues raised by these scenarios.

Finally, we remark that many in the AI community consider the presence of self and consciousness as the ultimate aim for strong AI (Tani, 1998; Prescott, 2015; Tegmark, 2017; Russell, 2019). We specifically do not aim for strong AI: we are not asking whether the machine possesses these human qualities. We instead only focus on how AI could be modeled and designed in order to better augment human capacities beyond their limitation – for that, the agent does not need to be conscious by itself and exhibit a sense of self (in the same way a vacuum cleaner does not need to be conscious to serve its purpose).

## Interlude – A View Into the Future

In this section, as inspiration, we sketch three scenarios in which human decision making could be augmented by an artificial agent that is sensitive to human interpersonal social and moral considerations. Most importantly, future artificial agents – of the kind we describe here – must continuously adapt to and be aligned with the prevalent social and physical econiche.

### Avoiding Moral Dilemma by Improving Decision Making

Imagine you are the chief executive officer (CEO) of a global coffeeshop company. You are looking for a personal assistant. That personal assistant should not only support you but, ideally, augment and thereby improve your decision making. Let’s sketch the following scenario: Due to a change in political climate in one country – that is a major market for your company – your business is targeted by protests against foreign imported coffeeshops. The situation is serious, with boycotts and violent demonstrations threatening your employees.

What do you do? One option would be to temporarily suspend all business in that country, ensuring the safety and comfort of your employees. That would incur severe short-term financial losses, however, and would probably close that country’s market in the future. A second option is to wait and see how the protests turn out and, more generally, how the political climate develops; while, at the same time, attempting to secure safety for the employees within the coffeeshops. Unlike in the first option, this would keep the country’s market open for the future. You are thus caught in a moral dilemma between human concerns and financial security.

Tools already exist that can (possibly with only mediocre accuracy) sketch the country’s development and the company shares on the stock market from the past to the present – to infer their near and far future. However, rarely does such a tool account for the economic and political factors in a way that is sensitive to the moral dimension in decision making. This is a missed opportunity; since, unlike humans, an artificial augmenting assistant would not get stuck in the vicissitudes of moral decisions that we as humans face on an almost daily basis.

Instead, a moral-decision-making assistant would be able to conceive a larger context, beyond the context we humans can perceive, by having access to thousands of case studies. This would allow integrating and reconciling the seemingly contradictory options, e.g., moral vs. financial imperatives in our case. In turn, this could guide and augment the CEO’s decision-making capacity, offering her the ability to more thoroughly perceive and reflect beyond the dichotomy of self-other – to a narrative that reconciles and integrates both perspectives on a deeper fundamental level in more global and long-term ways. Finally, legal constraints should also be taken into consideration, while the human relies on the assistant’s input. The accountability and legal responsibility of artificial assistants is an open question and current legal research tries to formulate principles for the assessment of such decisions and the consequences these might have.

### Predicting Natural Disasters

Now let us shift from economy to nature, invoking another scenario. We are living in a world full of catastrophic crises that involve cascades of events in a hierarchy of different time and space scales; these include wildfires, seismic earth waves with earthquakes, flooding, pandemics and storms (Friston et al., 2020; Scalabrini et al., 2020). Especially in the age of climate change, we require tools to properly predict such environmental crises well ahead of time in a more fine-grained way.

Let us take the recent example of the 2019–2020 Australian wildfires. To understand the instances and progression of the fires, effectors occurring at temporal and spatial scales that vary by several orders of magnitude should be considered. For example, the (positive) Indian Ocean Dipole (pIOD)– that characterizes high sea surface temperatures in the eastern and low sea surface temperatures in the western Indian Ocean, produces abnormal easterly winds that induce dry conditions in Australia and eastern Asia. These extreme events used to occur with a periodicity of approximately 20 years; however, non-linear models that incorporate greenhouse gas effects predict increasingly extreme pIOD frequencies. Wildfire prediction could thus incorporate these variables over a long temporal range that, as such, is not accessible to humans. Additionally, to construct regional predictions, daily (infrared) satellite imagery used to identify burnt areas would be enormously informative.

In other words, distinct data sources with varying predictive validity (e.g., on fuel and fire conditions) would be required to predict the occurrence and trajectory of such events. This kind of prospective inference goes far beyond human capacities. Even current AI methods, such as deep learning, which have been used for specific classification tasks (e.g., of burnt vs. not burnt earth) do not model the range of time-scales that would be needed to incorporate data portending a cascade of pre-fire changes. An artificial agent that *does* incorporate a rich and adaptive range of time-scales in its (generative) models of the future may, on the other hand, offer new promise.

This artificial agent may not only extend the frequency range of its sensorium – beyond humans – to ultraslow frequencies but also align with dynamics on a near continuous-time range of scales. That could enable it to detect when a chain of micro-events transition in a non-linear way into a prolonged period of bushfires. Analogously, we can potentially develop artificial agents that augment and ultimately enhance our perception of other environmental crises and their cascading antecedents in a progressively fine-grained spatial and temporal fashion. Please see Friston et al. (2017), for a worked example of this kind of hierarchical forecasting in the context of computational psychiatry and (Friston et al., 2020) for the coronavirus pandemic of 2020.

### Recovering Subjects’ Poor Alignment to Improve Their Mental Health

Finally, let us move from nature to human disease, most notably psychiatric disorders like schizophrenia and depression. In such disorders, an aberrant alignment of the subject to their environmental context has been implicated in major behavioral, cognitive, and mental changes (Northoff and Stanghellini, 2016; Northoff and Huang, 2017; Northoff, 2018b). For instance, depressed patients are locked within their inner cognitions, without being able to reach out to the outer world, rendering them isolated, sad and hopeless; i.e., depressed (Northoff, 2016) – an alignment that might otherwise have been adaptive in another setting (Badcock et al., 2017). While likewise schizophrenic patients are unable to synchronize with their environment, for instance, to music (Koelsch et al., 2019), but also their social and physical surroundings. This can lead to false inference and aberrant beliefs; i.e., delusions, and hallucinations (Parnas, 2012; Adams et al., 2013; Lakatos et al., 2013; Northoff and Duncan, 2016; Powers et al., 2017; Benrimoh et al., 2018; Parr et al., 2018).

Imagine now an augmenting agent that could help recalibrate these subjects’ alignment to their environment. Like a dance teacher who teaches you the rhythm of the music and how to dance to it, such an augmenting agent would “teach” these subjects’ brains to better synchronize with their social, cultural, and ecological contexts. The patient’s inner cognition may then be re-attuned to the events in the world, enabling them to re-engage and experience themselves as integrated rather than remaining isolated. At the same time, the schizophrenic patients’ brain would regain its capacity for alignment and synchronization (Tschacher et al., 2017), such that their inner beliefs and perceptions are better reflections of their lived world, i.e., attuned, with the outer reality. The patients’ pathological creation of an inner world, i.e., hallucinations and delusions, would revert to veridical inferences about what is actually going on “out there.”

Conceived in a wider context, beyond mental disorders, regaining a sense of agency – in other words, a sense of controlling one’s destiny – is important for mental health in general. Also, with the advent of modern brain computer interfaces (BCI) and other technologies, e.g., virtual reality, etc., achieving this re-alignment is not a distant goal anymore. It is now possible to build technologies that react directly to brain and bodily inputs – and send information to them to induce altered brain states.

## Artificial Intelligence and Environment – Learning From the Brain’s Adaptive Capacities

### Current Artificial Intelligence and Opportunities for Progress

Despite their differences, all three examples share the same basic theme. By extending the human capacities of decision making, temporo-spatial prediction (as in wildfires), and alignment (as in psychiatric disorders), the artificial agents here conceived augment the engagement and control of human agents. They do this by enabling a better interface with their respective environmental context, that is, a more tightly interwoven “environment-agent nexus,” and one which covers a broader temporo-spatial interface with the environment than humans. This may, in turn, extend the artificial agent’s capacities, i.e., decision making, prediction, and alignment, beyond those of humans.

The development of AI agents with near-human or super-human performance on some tasks has so far been driven by the paradigms of deep learning and reinforcement learning [see overviews in Tegmark (2017) and Russell (2019)]. Deep learning pioneers Bengio, Hinton and LeCun were honored with the 2019 Turing Award for their seminal work, while reinforcement learning, often combined with deep learning, has enabled recent high profile machine learning successes such as AlphaGo. Themes similar to those we propose, such as generative modeling of the world in reinforcement learning (Ha and Schmidhuber, 2018) or compositionality and hierarchicality (Han et al., 2020), have also appeared and been incorporated into reinforcement learning. In a much more top-down fashion, even models of an artificial self; i.e., self-consciousness, have been proposed and implemented, for instance by Tani (1998, 2016), Tani et al. (2008), Prescott (2015), and Prescott and Camilleri (2019).

However, despite all this progress, there is very little focus on the kind of artificial agents that might augment human selves in their moral decision making, or indeed appreciate their situated context. Augmenting human capacities requires the agent to be adaptive and thus align to the continuously changing environmental contexts of humans. This is well reflected in our three examples, where the agent’s relation and alignment to the environment is crucial for its ability to augment human capacities. The term “environment” is here meant to include cultural, social, natural, ecological, and geographic contexts.

Alignment, signifying adaptation to – and shaping by – the environment, then includes the long-term experience-dependence of the agent’s inner structure on her respective cultural and evolutionary context (Brown and Brüne, 2012; Heyes and Frith, 2014; Heyes, 2018; Veissière et al., 2019; Constant et al., 2020). Such shaping of the agent’s inner structure by its environment remains to be modeled in current artificial agents. Therefore, recent calls have been made from both within (Ha and Schmidhuber, 2018; Iwahashi, 2019; Russell, 2019) and without (Metzinger, 2018) AI, to improve the artificial agent’s interface with their respective environmental contexts. A dynamic and continuously adaptative interface with the environment (including its social, cultural, ecological, geographical, evolutionary, and other features) is not yet well-developed in AI at its current state.

### Aim of This Paper – Model for More Adaptive Artificial Intelligence

Our focus is on improving the agent’s adaptive interface with the environment. That would not only allow for developing artificial agents that could augment the capacities of human agents but would also be “trustworthy” (Tegmark, 2017; Russell, 2019). In the next section, we propose a novel AI approach.

We aim to learn from the brain. While much of current AI takes inspiration from the brain, we here focus on one specific aspect of the brain: its remarkable capacity to adapt and align itself to continuously changing environmental contexts. Motivating our focus is the fact that such rich adaptivity is an essential feature of agents augmenting human capacities along the lines of our three examples.

To enable rich adaptivity, we take inspiration from the principles by which the brain aligns itself to its continuously changing environments, both social and ecological. Recent neuroscience has brought to the fore two principles that drive adaptivity of an agent within its econiche – the free energy principle (FEP) and temporo-spatial dynamics (TSD). We propose that future AI methodologies may benefit from modeling artificial agents along the lines of FEP and TSD in order to create a rich environment-agent nexus that could augment human selves along the lines pictured above.

## Environment-Brain Interface – Conjoining the Free Energy Principle and Temporo-Spatial Dynamics

### Free Energy Principle (FEP) – Gradient Flow on Variational Free Energy Between Brain and Environment

Technically, the free energy principle casts neuronal dynamics as a gradient flow on a quantity known as *variational free energy* in Bayesian statistics and an *evidence lower bound* (ELBO) in machine learning. In this sense, free energy provides a specific cost function for optimizing over possible dynamics. But, moreover, the principle of FEP crucially focuses on variational free energy of the *pair* of agent- and environment- dynamics, and its minimization drives these to align. This can be seen in equation (1) where minimizing free energy will maximize the joint distribution of the brain’s sensory states (_*s*_) and hidden states of the environment (_ψ)_ (negative energy term), while simultaneously ensuring that the representation of the environment in the brain is maximally entropic (entropy term), therefore accommodating the most variable state of affairs possible within that distribution, in accord with Jayne’s maximum entropy principle:


(1) $$F\,\left(s,\mu \right)=E_{q}\,\left[-l\,n\,p\,\left(s,\psi |m\right)\right]-H\,\left[q\,\left(\psi \right)\right]$$

This furnishes a formal theory of active inference in the brain; sometimes referred to as *self-evidencing* (Hohwy, 2016).

In Eq. 1 the states s and _ψ_ are assumed to belong to *state spaces*, which could be any mathematical spaces where differentiation (computing a gradient) is possible, but it is common for them to be Euclidean space of some dimension. What’s more interesting is what a state represents: As typical in physics, machine learning, game theory, etc., a state is assumed to record all relevant information that characterizes an agent/environment in the present so its evolution in time can be determined. In our specific setting, the core of our proposal is to use FEP on TSD and so we wish to consider states that summarize temporo-spatial dynamics of relevance: for example, a musical instrument may be vibrating at a certain fundamental frequency with various overtones and moreover different parts of the instrument may be exhibiting different versions of this. The whole summary of all these dynamics in the present moment would constitute the instrument’s TSD state. Likewise, one could summarize a single instrument by less detail but also specify TSD for other instruments thus giving rise to the TSD state of an ensemble of musicians’ instruments and so on. Similarly, the various frequencies of brain activity in different brain regions could constitute the relevant state of a human brain. Of course, not all infinite detail is specified. We are interested in tapping into certain aspects of the “agent” and “environment” whose evolution we then describe with FEP. Recall that the agent can act on the environment to make it more aligned with the agent, or vice versa the agent can adjust its state to be better aligned with the environment. The intricacy of human behavior involves both modalities.

**FIGURE 1:**
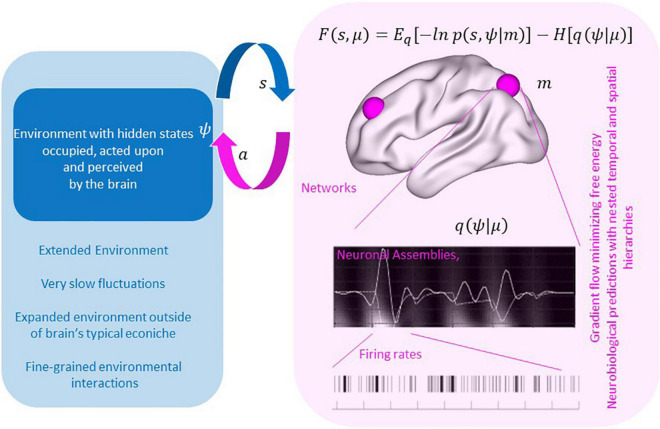
By minimizing free energy, brain and environment align as seen with the maximization of the joint distribution of the brain’s sensory states (_*s*_) and hidden states of the environment (_ψ_) while simultaneously ensuring that the representation of the environment in the brain is maximally entropic (entropy term). Currently the theory has considered neurobiological implementations of this gradient flows – resulting in testable imaging and electrophysiological predictions. With augmentation the goal may be to facilitate extended environmental states (lower left blue panel) that are not readily accommodated in neural states currently but could be accommodated in an artificial agent with extended “sensory” inputs.

Self-evidencing means that the brain refers to its own internal state, i.e., its intrinsic temporo-spatial dynamics, rather than exclusively to the external environmental events when making inferences about the causes of sensory inputs. This is essential to survival, but at the same time, in pathological instances, can lead to false inferences about states of affairs beyond the sensorium. For instance, many psychiatric disorders can be cast in terms of aberrant beliefs where subjects infer something is there when it is not (e.g., hallucinations) or infer something is not there when it is (e.g., an agnosia or dissociative disorder) (Edwards et al., 2012; Benrimoh et al., 2018; Figure 1).

Active inference is the core process that describes the environment-brain interface, namely, its interactive nature. That environment-brain interaction, i.e., the interaction of organism/brain and world/environmental context, can be characterized by free energy and, more specifically, variational free energy (Friston, 2010). Variational free energy is related to the discrepancy between the outside world and how an agent models and predicts that world. See (Friston and Frith, 2015) for an illustration of the implicit synchronization using simulations of birdsong and gradient flows on variational free energy.

Central to this concept is the notion of a generative model (*m*, in Eq. 1 above); namely an internal model embodied in the organisms’ brain. Due to its hierarchically organized temporo-spatial dynamics (see below for details), the brain can, effectively, perform gradient descent on the variational free energy within its respective environmental context. Taken in a more general way, variational free energy measures the degree of synchronization, alignment or attunement between the brain’s internal temporo-spatial dynamics and the environment’s external states that underwrite the former’s sensorium.

Neuronal dynamics can thus be described succinctly as gradient flow (i.e., descent) on variational free energy. These are necessarily approximated by any self-organizing system that can be distinguished from its environment (in virtue of possessing a Markov blanket) (Friston, 2013). For instance, the connection to perception (i.e., perceptual inference) rests on noting that a gradient flow on variational free energy is formally equivalent to a gradient flow on the logarithm of evidence for a model of the environment, entailed by the hierarchical brain; hence self-evidencing. Preempting later parts of our paper, the conclusion here is that hierarchical, (diachronic) temporo-spatial dynamics must characterize any augmenting AI that is modeling the same environment that we – as human selves – populate.

### Temporo-Spatial Dynamics (TSD) – Brain as Small-Scale Temporo-Spatial Model of the Environment

Using variational free energy to align to and model its environmental econiche, i.e., the respective environmental context, enables the brain to constitute mental features and functions. In fact, the free energy principle has already been used as a powerful formalism for modeling and understanding diverse mental features, including consciousness and affect/emotion (Gu et al., 2013; Seth and Friston, 2016; Clark et al., 2018; Smith et al., 2019a,b). Prominent in these studies is the application of free energy to the self as well as to different facets of self like the dynamic self, the bodily self and the subjective self (“I” vs. “me”) (Gallagher and Daly, 2018; Seth and Tsakiris, 2018), which all can be subsumed under the umbrella notion of “spatiotemporal self” (Northoff and Stanghellini, 2016; Northoff and Huang, 2017).

Importantly, the link of free energy to mental features like self can be predicated on temporo-spatial dynamics; as for instance in “deep temporal models” that possess a necessary *temporal thickness* or depth (Seth, 2015). It has been argued that a necessary characteristic of generative models that support consciousness and intentionality is precisely their capacity to model the future (Friston, 2018). These “deep temporal models” are thus crucial for the human to adjust and thus align to the ongoing temporal dynamics of their environment. This results in a deeply temporal environment-agent nexus that, as such, naturally conjoins variational free energy and temporo-spatial dynamics.

Furthermore, the very nature of free energy minimization – as tuning a generative model to a hierarchical or deep world with separation of temporal scales – necessarily means that hierarchical temporo-spatial dynamics must be recapitulated in any such aligning or adapting agent. In the language of self-organization, this is what has been described as “good regulator theorem” that describes the intimate model-like relationship between the regulator of a system and the regulated system: “every good regulator of a system must be a model of that system” (Conant and Ross Ashby, 1970; Seth, 2014, 2015).

Specifically, this means that the environmental hierarchies of different events may be recapitulated and thus modeled by the brain itself within its own intrinsic hierarchical organization, i.e., its temporo-spatial hierarchy. There is no need for the living to represent a model of the environment in their head: “An agent does not *have* a model of its world – it *is* a model. In other words, the form, structure, and states of our embodied brains do not *contain* a model of the sensorium – they *are* that model.” (Friston, 2013). Such modeling of the environment by the brain is driven by the need of the brain to minimize its variational free energy with its respective the environmental context.

The brain can be conceived as a free energy-driven temporo-spatial model of its environmental hierarchies. That results in temporal and spatial nestedness of the brain within its respective environmental context. Despite different temporal (and spatial) scales across body, brain, and environments, they are nevertheless connected through self-similarity in their shape or form. Just like the smaller Russian doll is contained within the larger one (same shape, different size), the brain and its temporo-spatial model nest in a self-similar way within the much larger environment. Given such self-similarity between brain and environment, we may better focus on “what our head’s inside of” rather than searching for “what inside our heads” (Bruineberg and Rietveld, 2014, 2019).

## Intrinsic Organization of the Brain – Spatial and Temporal Hierarchies

Driven by its variational free energy with the external environment, the brain is a model of the temporo-spatial dynamic of the latter within its own internal organization. This raises the question of the nature of the brain’s detailed spatial and temporal features of its intrinsic organization – that shall be the focus in the following.

### The Brain’s Intrinsic Spatial Organization –Core-Periphery Structure

**FIGURE 2:**
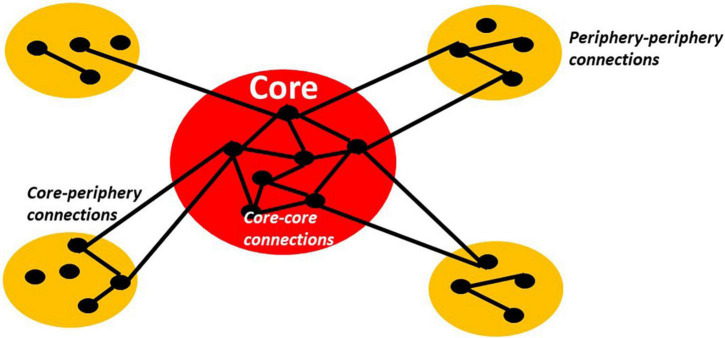
The schema shows typical core-periphery organization with high connectivity/relationship (lines between dots) among the nodes or members (dots) of the core as well as low connectivity/relationship among the nodes of the periphery and between core and periphery nodes.

What kind of hierarchical organization should be implemented in our artificial agents? We suggest again to look to the brain and its temporo-spatial hierarchical organization. Different models, relying on distinct principles, have been suggested for the cortical organization (Markov et al., 2013). Being based strongly on anatomical grounds, medial-lateral and especially rostral-caudal models as well as modular models have been proposed for the human brain (see Margulies et al., 2016; Huntenburg et al., 2018 for excellent discussions). The rostral-caudal model suggests an anatomical gradient from more unimodal subcortical and sensory regions to more heteromodal prefrontal regions, which can be distinguished by their micro- and macro-structural/architectonic features (Northoff, 2010; Northoff et al., 2011; Gollo et al., 2015, 2017; Margulies et al., 2016; Huntenburg et al., 2018). However, more recent, functionally oriented, investigations question the primacy of such rostral-caudal organization (see Figure 2).

Margulies et al. (see also Bassett et al., 2013; Huntenburg et al., 2017, 2018) suggest rather an onion-like model of the human brain, featuring different, i.e., inner, middle, and outer, layers. Inner layers mediate *trans*-modal internally oriented functions like self (Northoff et al., 2006), episodic simulation (Schacter et al., 2012), and mind wandering (Christoff et al., 2016). Despite their differences, these distinct forms of internal cognition all strongly recruit the default-mode network (DMN) that is situated at the core in the brain’s overall spatial organization (Margulies et al., 2016; Huntenburg et al., 2018). In contrast, unimodal functions like motor and various sensory modalities implicate sensorimotor cortices that represent the outer layers, e.g., the periphery (see also Northoff, 2011; Northoff et al., 2011).

The onion-like model entails the distinction between a core, e.g., the most inner layer, and a periphery, e.g., the outer layers. This amounts to what has been described as “core-periphery model” in social science (Borgatti and Everett, 2000) and the centripetal hierarchies proposed by Mesulam in neurobiology (Mesulam, 1998). A centripetal or core-periphery architecture can be characterized by a core that shares nodes with strong interconnections among each other. These core-core connections are much stronger than the connections of the core to the periphery, e.g., core-periphery connections, and also the connections among the nodes within the periphery itself, e.g., periphery-periphery connections (Borgatti and Everett, 2000). Such core-periphery has been shown to also apply to the brain (Margulies et al., 2016; Huntenburg et al., 2017, 2018; Gu et al., 2019) and akin to other models like “rich club” (van den Heuvel and Sporns, 2013), “dynamic core” (Tononi and Edelman, 1998; de Pasquale et al., 2012, 2016, 2018), and “global workspace” (Dehaene et al., 1998, 2017; Mashour et al., 2020).

### The Brain’s Intrinsic Temporal Organization–Temporal Hierarchy

How about the brain’s intrinsic temporal organization? Hasson and colleagues conducted a series of fMRI studies of the encoding of external stimulus sequences (music, movies, etc.), where stimuli (words, sentences, paragraphs, etc.) had different durations – short, medium, and long (Honey et al., 2012; Chen et al., 2015, 2017; Hasson et al., 2015). Using inter-subject correlation of task-evoked fMRI data, they associated stimulus duration with responses in different regions. This enabled them to infer that the different regions exhibit different degrees or windows of temporal integration for encoding and receiving external stimuli –cast in terms of “temporal receptive windows” (TRW; see Lerner et al., 2011; Hasson et al., 2015; Simony et al., 2016).

Specifically, they observed that words (1 s −/+ 0.5 s) elicited activation in lower-order primary sensory regions like visual (when presented visually) or auditory (when presented auditorily) cortex. Sentences, lasting longer, (8 +/− 3 s) were associated with higher-order regions like medial temporal and parietal cortex. In contrast, whole paragraphs lasting about (38 +/− 17 s) recruited activity in the DMN (see Honey et al., 2012; Stephens et al., 2013; Hasson et al., 2015; Simony et al., 2016).

Together, these data show that different regions exhibit different durations in their TRW’s and thus different time scales during task-related activity, suggesting a certain temporal hierarchy (Hasson et al., 2015), that may mimic the centripetal spatial organization. This is related to (i) the externally presented stimuli, (ii) the brain’s own internal spontaneous activity (as measured in the resting state), and (iii) the brain’s spatial core-periphery organization.

**FIGURE 3:**
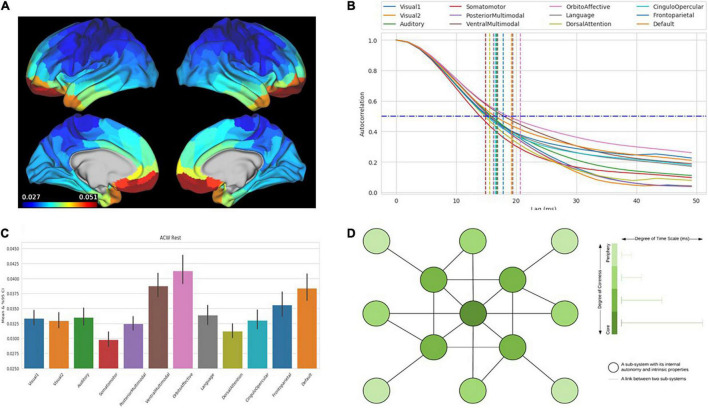
The figures show the intrinsic neural timescales in the human brain calculated by autocorrelation window (ACW) in human brain data from Human Connectome data set (MEG). **(A)** Distribution of the duration of intrinsic neural timescales duration throughout the brain and its different regions (red: longer duration of ACW). **(B)** Decrease in autocorrelation (*y*-axis) of neural activity over time (*x*-axis) in different networks (different lines) with demarcation at 50% (ACW) (vertical lines). **(C)** Values of ACW (*y*-axis) for the different networks (*x*-axis). **(D)** Schematic depiction of core-periphery organization with the color shading of the nodes reflecting the length of intrinsic neural timescales (more dark = longer timescales, more light = shorter timescales).

(i) These data show that the brain’s time scales are directly related to the time scales of stimulus- bound responses. This entails some form of temporal correspondence of the brain’s internal neuronal dynamics during task-related activity in both lower- and higher-order sensory and cognitive regions with the temporal structure of the external environmental stimuli and events. One can thus conceive the brain’s time scales, i.e., its TRW, as one manifestation of the brain’s alignment to its environment in that the brain’s internal temporal hierarchy is matched to that of the external environment.

(ii) Most interestingly, a more or less analogous hierarchy of time scales can be observed not only during evoked activity but also in the brain’s spontaneous or intrinsic activity (Murray et al., 2014; Chaudhuri et al., 2015; Gollo et al., 2015, 2017). Measured by the correlation between different time points of neural activity, i.e., the autocorrelation window (ACW), these studies demonstrate diverse correlation lengths, (i.e., ACW), in different regions, in the resting state. Pending more robust results about the temporal hierarchy in the brain’s resting state, these data suggest that the hierarchy of time scales is an intrinsic feature of the brain itself and not just shaped by the external task itself. Building on the previous part, we assume that the brain’s hierarchy of intrinsic time scales can be conceived in a much broader way. The brain’s hierarchy of intrinsic time scales does not model but is by itself a small-scale self-similar miniature model of the larger-scale environmental hierarchies themselves, including their historically and evolutionarily shaped features (Figure 3) as mirrored in the brain’s current and past experience of these.

(iii) Finally, one may want to raise the question how such a hierarchy of intrinsic time scales maps onto the brain’s spatial organization in a core-periphery architecture. The different time scales and implicit temporal hierarchy operates across the functional anatomy described above. The regions in the core show rather long ACW and thus more extended intrinsic neuronal time scales than the periphery, where the ACW is relatively shorter (Murray et al., 2014; Chaudhuri et al., 2015; Gollo et al., 2015, 2017).

Specifically, Gollo et al. (2015) show that core regions; e.g., rich club regions like DMN and the insula evince predominantly slow time scales, with stronger power in infra-slow (0.01 to 0.1 Hz) and slower (0.1 to 1 Hz) and relatively weaker power in faster frequencies (1–180 Hz; see also He, 2011; Huang et al., 2015, 2016; Zhang et al., 2018; Wolff et al., 2019a). In contrast to the DMN as core, sensorimotor regions in the outer periphery exhibit relatively less power in the infra-slow and slow frequency ranges – and relatively more power in the faster ranges (He, 2011; Huang et al., 2015).

Together, this amounts to an intricate temporal or dynamic hierarchy; i.e., a chronoarchitecture within which each region is featured by its “natural frequency” or “intrinsic neural time scale” (Bartels and Zeki, 2005; Kiebel et al., 2008; Honey et al., 2012; Gollo et al., 2015, 2017; see Figure 3). More generally, the brain’s intrinsic temporo-spatial hierarchical organization, as we have seen, can be conceived as self-similar miniature model of the temporo-spatial complexities of its environment – albeit in a much smaller and more compressed scale, as manifest in our perception and cognition of that very same environment (Northoff, 2018a).

## Modeling the Environment-Agent Nexus Using Free Energy Principle and Temporo-Spatial

### Artificial Agents – Modeling a Free Energy-Driven Intrinsic Temporo-Spatial Hierarchy

In the above, we laid the groundwork for a mathematical formalism and showed the structural organization of the brain’s highly adaptive capacities. Such a mathematical formalism may furnish the foundation for developing a highly adaptive and thus “aligning” artificial agent as a first step toward next generation AI paradigms. Specifically, the mathematical formalism we proposed integrates the free energy principle (FEP) and temporo-spatial dynamics (TSD), and could be employed by agents in their computational algorithms. This will enable a delicate balancing of dynamical stability and adaptability between agent and environment – as it is central for applying AI to augment human perception and cognition (see Russell, 2019, for a review of some recent AGI developments in this direction). We now turn to modeling questions, in the AI sense of the word, where a model is a part of a learning algorithm that mimics whatever the agent will try to optimize. If the agent is tasked with learning to identify photos of cats and dog with as few errors as possible then the situation is relatively simple: it must search in a specific space of mappings from photos to the labels cat/dog. This is the “model.” It may for example be represented as sides of a hyperplane in a high-dimensional space (a feature space) that photos are mapped to. If the agent is a robot that is tasked with moving around an uncertain terrain it may model a 4-legged animal, in that it has metal extensions that can be moved with motors, and the various possible movements in response to sensory input from the terrain would also be part of the model.

If we now want to develop a highly adaptive, “aligning” artificial agent as a first step toward next generation AI that mimics the way a human aligns then one core feature is the modeling of an intrinsic temporo-spatial hierarchy in the agent. Future AI models may want to implement such intrinsic spatial and temporal organization in their artificial agents, including the different time scales and the core-periphery organization [see Yamashita and Tani, 2008 – for first steps in this direction in artificial agents using what they describe as “multiple time scale recurrent neural network,” (Paine and Tani, 2005; Tani, 2016); as well as (Choi and Tani, 2017) who emphasize the need for temporal hierarchies in artificial agents for their adaptation to the environment]. Spatiotemporal hierarchies would extend the current – often module-based – models of artificial agents (Prescott and Camilleri, 2019) to the above mentioned core-periphery organization. The core-periphery organization could be complemented by combining top-down (providing the agent’s inner input) and bottom-up (providing the agent’s outer input) layers – that Tani uses in his compelling model of an artificial agent (Tani, 1998; Tani et al., 2008; Choi and Tani, 2017; see also Iwahashi, 2019).

Most importantly, by conjoining it with FEP, the artificial agent’s intrinsic temporo-spatial hierarchy may be a small-scale but self-similar model of its own environmental context. To achieve that, the agent’s temporo-spatial hierarchy needs to be highly dynamic and continuously changing, so as to adapt to the changing environmental dynamics. More specifically, this means that the causal (or temporo-spatial) architecture of the environment must be recapitulated or installed in the agent’s temporo-spatial dynamics in such way as to allow the agent to minimize its variational free energy with its respective environmental context.

### Augmenting Agent Meets Augmented Agent – Adapting the Agents’ Temporo-Spatial Dynamic to Augment Humans

How can such an artificial agent augment human capacity along the lines of our three future scenarios? We suppose that free energy-driven intrinsic temporo-spatial organization provides the artificial agent with the kind of balance between temporal stability and adaptivity (Kiebel et al., 2009; Friston et al., 2012) that is essential for its role in its environmental context. The artificial agent will consequently be highly dynamic and stable at one and the same time. In turn, this enables the agent to interact with humans that can be characterized by a somewhat analogous hybrid of temporal dynamic and stability. The artificial agent could then, to a certain degree, mirror the humans and, even stronger, synchronize with them in a temporo-spatial way that is necessary for any form of dyadic exchange or communication (see Friston and Frith, 2015 for empirical support and numerical analyses).

A similar theme emerges in the context of human-agent interactions, known as “human in the loop.” In this setting, artificial agents generate enormous amounts of information regarding complicated problems aided by human input before reaching a final decision (see Edelman et al., 2019 for robotics). In this setting, agents rely on expert humans to adjust errors in their intermediate predictions; thus, the accuracy of the algorithm increases.

So, how can we construct an artificial agent’s intrinsic temporo-spatial organization to ensure it not just mirrors but truly augments human selves? Based on the conjoining of FEP and TSD, one can expose the artificial agents to different simulated social and natural or ecological environments with, for instance, a broader and more fine-grained frequency range beyond the one available through the sensory epithelia of one human. That, as we suppose, should extend the artificial agent’s spatial and temporal organization beyond the one of humans to, for instance, a wider and more fine-grained range in the power spectrum.

How can such extension of the artificial agent’s interface with its environmental context beyond the ones of humans facilitate its interaction with the humans themselves? This is where the free energy principle comes in. Since variational free energy is an extensive quantity, the augmenting and augmented agents will, following an information theoretic measure of augmentation, minimize their joint free energy (Bruineberg and Rietveld, 2014; Friston and Frith, 2015; Constant et al., 2020). In turn, this will inevitably lead to a generalized synchrony between the augmenting (AI) and augmented (human) agent.

## Can Artificial Agents Augment Humans – Coming Back to Our Examples

### Enhancing Moral Decision Making – Self- Other Continuum in Decision Making and “Trustworthy AI”

Humans are able to change their belief updating and contextualize their objectives. That is, for instance, manifest in our decision making that operates on a balance between environmental constraints; i.e., externally guided, and self- or ego-centric concerns, i.e., internally guided (Nakao et al., 2012, 2013, 2016, 2018, 2019; Wolff et al., 2019a,b). If the subjective preference dominates, as in the choice of a certain moral values or a specific profession (independent of objective values), internally guided decision making dominates.

In contrast, if one assesses the external input according to purely objective (rather than subjective) criteria, externally guided decision making dominates. Technically, the balance between adjudicating between internal and external preferences can be articulated in terms of the confidence placed in – or precision afforded to – prior beliefs about the sorts of outcomes that follow “good” and morally valuable decisions (Friston, 2013).

Moral decision making paradigmatically exposes the internal-external continuum, i.e., self-other continuum. For instance, the well-known footbridge dilemma raises the question whether one is willing to sacrifice one’s own life in favor of sacrificing the life of several other people (Wolff et al., 2019a). Who is more important – the own self or the other self? In our first example, the CEO is caught in such moral dilemma, that is, between more self-or ego-centric concerns of the company and the wellbeing of the employees – one can thus speak of a self-other continuum in our decision making.

An optimal artificial agent would consider both self and other, as in our very human decision making. And, even more important, it would allow reconciling and integrating both at a deeper more fundamental level which escapes us as humans (for which reason we are trapped in moral dilemmas like the footbridge dilemma). Current AI does not significantly address the self-other continuum and the deeper more fundamental level of their integration. Even though the name “value function” is very explicitly a function that serves as a surrogate for moral values, how are these fluidly to be combined with external criteria? Some approaches exist: e.g., Dayan and Hinton (1992), which was motivated by speeding up reinforcement learning (RL) with a control hierarchy. But this architecture itself is hard-coded and imposed by the designer, rather than seeking any reciprocity with the environment. Another approach is suggested by Naruse et al. (2018) based on category theory, where the values of different sub-agents are in principle aggregated.

Novel paradigms – such as the one suggested here – may have other mechanisms for incorporating diverse criteria, i.e., internal, and external, into decision making. In particular, active inference formulates value in terms of prior preferences that are internal preferences of the system itself. These internal preferences are effectively (sub personal or non-propositional) Bayesian beliefs about the consequences of action. Crucially, this means that internal preferences and implicit value are attributes of beliefs about *anything* – and cannot be reduced to a single value or an external fact. Importantly, these internal preferences can by themselves be traced to the agent’s relationship with her/his past and present environmental context – it is that very same environment-agent alignment that endows and constitutes the value of these internal preferences, including their potential moral values (Northoff, 2018a).

At the end of the day, regardless of the architecture, we care about the artificial agent’s alignment and how well it can incorporate the self-other continuum and, going beyond humans, integrate this on a deeper more fundamental level. Another way of expressing this is to estimate an agent’s prior internal preferences that are implicit in its decisions and choices about external conflicts. Indeed, this approach has been adopted formally through the notion of computational phenotyping (Schwartenbeck and Friston, 2016). Ultimately, the agent’s expanded interface with the human agent’s environment may allow the artificial agent to not only develop moral values acceptable to humans but, even more, become an example of “trustworthy AI” (European Commission [EC], 2019) (and ideally to be more “trustworthy” than human agents), as discussed extensively in policy-making and research circles (European Commission [EC], 2018; Floridi et al., 2018; Metzinger, 2018; Veale et al., 2018; Castelo, 2019; European Commission [EC], 2019; Salles et al., 2020).

### Predicting Natural Disaster – Augmenting Complexity Matching of Environment and Brain

One instance of testing metrics of alignment of an agent to its environment consists of what has been described as “complexity matching” (Kello et al., 2010; Borges et al., 2018). Briefly, complexity matching allows to compare, i.e., correlate and match the degrees of spatial extensions and/or time scales between two different systems (Salvanes et al., 2013; de Pasquale et al., 2016; Borges et al., 2018). The degree of complexity matching between brain and environment is considerably enhanced by the fact that the brain’s temporo-spatial structure including its hierarchy is strongly shaped by the environment and its different time scales including both life-span and evolutionary timescales – this amounts to experience-dependence (as understood in a wide sense as exposure).

Accordingly, by letting itself and its temporo-spatial hierarchy be shaped by its environmental context, the brain increases its likelihood of higher degrees in the matching of its own temporo-spatial complexities with the ones of its environmental context. In particular, complexity matching of brain and environment is inherently temporo-spatial and therefore strongly dependent upon the brain’s temporo-spatial hierarchy. What AI describes as dynamic adaptation (Tani, 2016; Ha and Schmidhuber, 2018; Han et al., 2020) is likewise related and may thus be traced to complexity matching.

However, current AI has problems in complexity matching with respect to heterogenous, highly variable and dynamic environments including social, cultural, and ecological – that is, for instance the case in our example of the wildfires where the ecological context continuously changes. This may, in part, be related to a rather constrained architecture in current artificial agents that allows only a limited range of timescales and consequently alignment with a rather restricted number of different environments. Moreover, modeling in brain-like artificial agents, i.e., “animats” as the authors say, demonstrates the experience-dependence (in the sense of exposure) of the agent’s internal structure as its inner complexity is dependent upon the complexity of the outer environment (Edlund et al., 2011; Joshi et al., 2013; Albantakis et al., 2014).

That is just a first step though; it leaves open the matching of environmental structure and the agent’s internal structure with the shaping of the latter by the former, i.e., complexity matching and “complexity shaping” as one may want to say. The realization of complexity matching, and temporo-spatial hierarchical organization can be considered a first step toward increasing the degree of the agent’s complexity matching with the environment. For that to be possible, the temporal range of the agent’s power spectrum and its spatial expansion of regions and networks may need to be extended beyond the ones of humans.

Such agents would, for instance, exhibit a larger range of different frequencies (than humans) which would allow them to better match with their environmental context in a broader and eventually more fine-grained way. The agent’s interface with its environmental context may thus be expanded which, leading to higher degrees of complexity matching of agent and environment, may be especially relevant for our second case, the prediction of natural disasters with cascading events prior to the actual outbreak.

### Alleviation of Symptoms in Mental Disorders – Brain-Computer Interface Modulating the Subject’s Environment-Brain Alignment

While we usually take our brain’s capacity for alignment to its environment for granted (as we do not explicitly perceive it), we are painfully aware when our brain’s alignment to its environmental context is not properly functioning anymore. That is, for instance, the case in coma where we, having lost completely our brain’s capacity of alignment, are no longer able to make any decisions and navigate in the environment (Zilio et al., 2021). Yet another instance of altered alignment are mental disorders like depression and schizophrenia – or indeed the use of psychedelics (Carhart-Harris et al., 2016).

**FIGURE 4:**
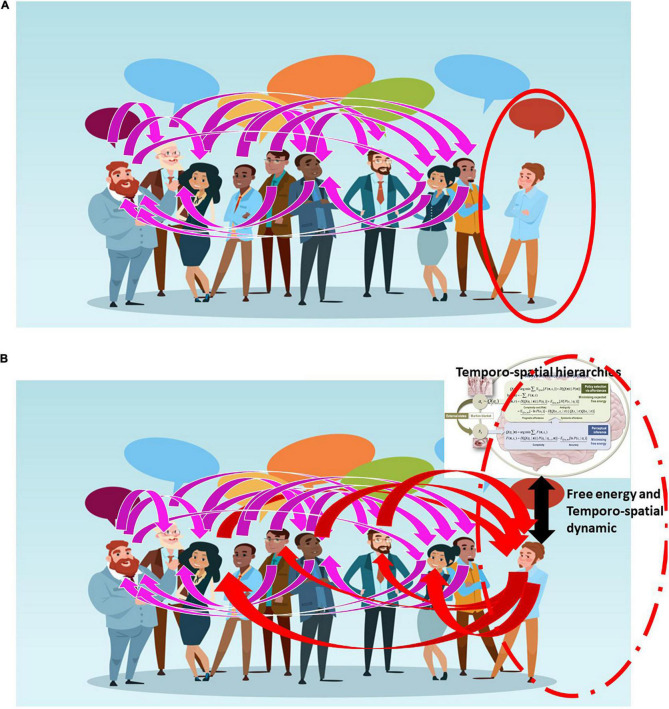
The figure depicts schematically the connection/relationship or alignment between different persons (magenta lines) with one exception as that person (on the right with red circle) is isolated from the others, being unable to connect or relate **(A)**. Improvement of that person’s alignment to others is possible by using the kind of adaptive models (upper right corner) we here suggest – this breaks the person’s isolation allowing them to connect and relate to others (red lines) **(B)**.

In the case of depression, one withdraws from the external environment, resulting in abnormally elevated internal focus; i.e., increased self-focus, at the expense of the environment-focus (Northoff, 2007, 2016). See Figure 4. The self-other continuum is here shifted abnormally toward the pole of the self – this leaves subjects with social withdrawal, negative mood, sadness, and suicidal ideation. While in schizophrenia, subjects lose their brain’s ability to align and synchronize with external stimuli (Lakatos et al., 2013; Northoff and Duncan, 2016). The internal-external continuum and thus the self-other continuum is here not only shifted but disrupted – that results in the perception and cognition of the external environment in terms of the own internal imagination and thoughts, i.e., hallucinations and delusions as typical symptoms of such inner-outer confusion (Parnas, 2012; Northoff and Duncan, 2016).

One central therapeutic aim in these patients entails “normalizing” their matching and thus their gradient flow on variational free energy with the environment, in the hope that they can re-align to the environment in a “normal” way (see Figure 4). As described in our third example, artificial agents may improve our current, rather limited, therapeutic tools in this respect. Specifically, one would like to construct an artificial agent that, implemented as a brain-computer interface (BCI), can (i) continuously record and monitor the individual brain’s alignment to its environmental context along the lines of internal-external self-other continuum; (ii) determine continuously the individual’s optimal and non-optimal degrees of alignment in relation to environmental context; and (iii) modulate the individual’s brain’s actual alignment by shifting it toward more optimal degrees in psychiatric patients; for example, those suffering from depression or schizophrenia. This may sound fanciful; however, treating simple things like tremor may yield to this sort of dynamical intervention (c.f., chaos control). Please see Cagnan et al. (2017) for a nice example.

Ideally, such an artificial agent may extend the capacity of therapists to determine optimal and non-optimal levels of alignment (approaching the capabilities of a healthy subject who senses and modulates his/her own environment-agent nexus). However, irrespective of the *method used* to achieve better environment-self alignment; improved wellbeing and alleviation of distress and discomfort associated with disorders remain the goals of a therapeutic AI tool like this. Will such augmenting agents thus exert true therapy? We may simply want to ask the users themselves, i.e., the augmented agent: does the tool improve your wellbeing and/or alleviate distress or discomfort? how does it compare to human-therapist intervention (if this has been tried)?

To test our hypothesis that conjoining of FEP and TSD is useful for designing IA’s of the three types listed here we propose that initial prototypes of such agents be subjected to two kinds of *litmus test*. First, direct tests of basic aspects of the desired alignment that are known to be relevant, such as complexity matching as described above. And second, tests that involve human ratings and can thus be seen as IA-analogs of the famous Turing test (Turing, 1950): do specific users rate a decision making tool as moral; do users from many cultures rate the tool as culturally sensitive; does the tool improve your wellbeing and/or alleviate distress or discomfort; how does it compare to human-therapist intervention (if this has been tried)?

## Conclusion

We propose a novel AI approach. Rather than creating human intelligence, we propose augmenting it. For that purpose, we suggest taking lessons from the brain as a key strategy in attempts to build artificial agents that can support and augment human selves. Taking lessons from the brain, we suggest that the artificial agents should first exhibit internally a complex, hierarchical temporo-spatial structure which, secondly, should be continuously shaped and updated through minimization of variational free energy within its respective environmental context.

The aim is to enable the continuous shaping and construction of the agent’s inner spatial and temporal organization in a hierarchical manner, driven by its gradient flow on variational free energy with the respective environmental context. That serves as basis for constructing both dynamic stability and adaptivity of the agent to its respective environmental context, namely, the human whose capacities it shall augment.

Such in-built environment-agent nexus will provide novel opportunities for AI as suggested in our initial examples. Even though it does not create and thus possess the human capacities by itself, the augmenting agent will nevertheless allow broadening our brain’s interface with its environment. That, in turn, has the potential to stabilize and hence our brain-based self especially in times of crisis like pandemics as well as to augment our perceptual and cognitive capacities beyond our current human limits.

## Data Availability Statement

The original contributions presented in this study are included in the article/supplementary material, further inquiries can be directed to the corresponding author.

## Author Contributions

KF supervised the project. KF and GN contributed expertise, respectively, in the Free Energy Principle and Temporo-Spatial Dynamics, while the combining of these in the present manuscript was conceived by KF, GN, and MF. JG, DP, PP, and RM contributed to the final manuscript. All authors contributed to the article and approved the submitted version.

## Conflict of Interest

The authors declare that the research was conducted in the absence of any commercial or financial relationships that could be construed as a potential conflict of interest.

## Publisher’s Note

All claims expressed in this article are solely those of the authors and do not necessarily represent those of their affiliated organizations, or those of the publisher, the editors and the reviewers. Any product that may be evaluated in this article, or claim that may be made by its manufacturer, is not guaranteed or endorsed by the publisher.
